# What is the cost benefit ratio of sending adeno-tonsillar tissues for histology: does adenoid/tonsillar tissue in children undergo malignant transformation?

**DOI:** 10.11604/pamj.2019.32.193.17142

**Published:** 2019-04-18

**Authors:** Josephat Maduabuchi Chinawa, Awoere Tamunosiki Chinawa, James Akpe, Lovelyn Kelvin-Iloafu, Vivian Onukwuli

**Affiliations:** 1 College of Medicine, Department of Pediatrics, University of Nigeria, University of Nigeria Teaching Hospital (UNTH), Ituku-Ozalla, Enugu State, Nigeria; 2 Enugu State University Teaching Hospital, Enugu, Nigeria; 3 Department of Ortholaryngology, University of Nigeria Teaching Hospital (UNTH), Ituku-Ozalla, Enugu State, Nigeria; 4 Department of Management, Faculty of Business Administration, University of Nigeria, Enugu Campus, Enugu State, Nigeria; 5 College of Medicine, Department of Pediatrics, University of Nigeria, University of Nigeria Teaching Hospital (UNTH), Ituku-Ozalla, Enugu State, Nigeria

**Keywords:** Adenoids, tonsils, children, histology

## Abstract

Due to the enormous amount spent on histology of adenoid and tonsillar samples from children with adeno-tonsillectomy with no confirmed result of malignancy, it has become expedient to reconsider sending such tissues for histologyThe objective of this study was to determine the necessity of sending tissues of adenoid and tonsils for histology by means of ascertaining the prevalence of malignancy among children with adeno-tonsillectomy. This was a retrospective study done in three private hospitals that provide care for children in Enugu. Data was obtained from the medical records of 72 patients who had undergone tonsillectomy and/or adenoidectomy from September 2011 to May 2018. All the surgical cases done had their samples sent immediately for histology. A total of 72 adeno-tonsillar tissues were taken for histology of which all showed lymphoid hyperplasia with none showing any form of malignancy. Age group ranged from 6 months-18 years with 57 males and 15 females. Histology of the adeno-tonsillar tissue specimen was done among all the children with each costing 9000 Naira (26 US dollars). There were 3 tonsillectomies, 1 adenectomy and 68 adeno-tonsillectomies done. Indications for surgery were mainly upper airway obstruction for 69 cases and recurrent tonsillectomy for 3 cases. Histology revealed lymphoid hyperplasia for all cases. None of the patients in our study had histologic evidence of malignancy. Routine histopathologic examination in adeno-tonsillectomy specimens among children may be dispensable as it showed a negative cost-benefit ratio.

## Introduction

Adenoid hypertrophy arises when there is a condensation of lymphoid tissue at the posterior-superior wall of nasopharynx [1]. It occurs as a physiological change in children between the age of 6-10 years but atrophies at the age of 16 years [2]. Literatures also show that adenoidal hypertrophy rarely indicates a malignant diagnosis [3-5]. Though it may be difficult to distinguish neoplastic adenoidal tissue from benign hypertrophy based on the macroscopic appearance alone. This forms the basis why many clinicians sometimes face the dilemma whether or not to biopsy adenoidal mass in children and adults [4, 5]. Care givers spend so much on histology of adenoid/tonsillar issue after paying a great deal on surgical removal which is more often from out of pocket. This study is therefore necessary to show if adenoid/tonsillar tissue histology is necessary after adeno-tonsillectomy by means of ascertaining prevalence of malignancy among children with adeno-tonsillectomy.

## Methods

The study was conducted in 3 private hospitals. The hospital provides care for children. All the surgical cases done had their samples sent immediately for histology. The subjects whose ages were between 6 months and 18 years were included in this study, while those subjects whose surgical intervention included adenoids and other tumours as leukaemia were excluded from the study. It is a retrospective study based on data obtained from medical records of 72 patients who had undergone tonsillectomy and/or adenoidectomy from September 2011 to May 2018 in all the private hospitals studied. All the children with adeno-tonsillar hypertrophy had adenoidectomy and/or tonsillectomy by curetting and dissection with snaring method respectively under general anaesthesia. Specimens were kept in sterile bottles with 10% formaldehyde and sent for histological analysis. The specimen was preserved in formalin for 24 hours and after dehydration, a histological procedure was done using paraffin dyed with hematoxillin-eosin and analysed microscopically.

## Results

A total of 72 adeno-tonsillar tissue were taken for histology of which all showed lymphoid hyperplasia with none showing any form of malignancy. Age group ranged from 6 months-18 years with 57 males and 15 females. Histology of the adeno-tonsillar tissue specimen was done among all the children with each costing 9000 Naira (26 US dollars). There were 3 tonsillectomies, 1 adenectomy and 68 adeno-tonsillectomies done. Indications for surgery is mainly upper airway obstruction for 69 cases and recurrent tonsillectomy for 3 cases (Table 1). Histology revealed lymphoid hyperplasia for all cases. None of the patients in our study had histologic evidence of malignancy.

**Table 1:** Results of histopathologic examination of the adenoids and tonsils

Exam result	Number of tonsils	Percentage
AT with LH	71	98.6
AT with LH and suppurative foci	1	1.4
AT with LH with areas of surface erosion	0	0
AT with LH with colonies of actinomyces	0	0
AT with LH and sub mucous fibrosis	0	0
AT with recent haemorrhage	0	0
Papilloma squamous cells	0	0
Total	72	72

**KEY:** AT: Adeno-tonsillitis, LH: lymphatic hyperplasia

## Discussion

Most otolaryngology services usually send tissue specimen from adenoids, tonsils or both for histopathologic examination. This they do mainly to rule out malignancy or as a routine investigation or even for medico-legal reasons [6]. Previous studies have shown that routine histopathologic analysis of the tonsil is unnecessary, because this histology review normally show extremely low prevalence of malignant changes [7]. Based on our study, it is obvious that histopathology of tissues from adeno-tonsillectomy is inexpedient, this is more so in our country with a high economic meltdown and a high rising foreign exchange. Our study showed a zero prevalence of malignancy in tissue extracted from adeno-tonsillectomy in children. This is akin to the studies of Giseli *et al.* [6] and Vema *et al.* [7] who obtained zero prevalence of malignant adenoid tissue and questioned the need for histology after adenoid and tonsillar surgery in children. Other literature though refuted our findings but yet show a very low prevalence of malignancy in adeno-tonsillar tissue. For instance, Randall *et al.* [8] reported a prevalence of malignancy of 0.087% in routine examinations. However, about 9 out of every 10 of his patients had preoperative suspicion of malignancy. Among his patients, he also noted that 0.011% had a positive result of malignancy without any risk factor. He then concluded that routine examination was unnecessary if there was no suspicion of malignancy. Furthermore, Garavello *et al.* [9] noted a 0.18% incidence of positive histopathologic analysis without clinical suspicion in children, concluding that routine examinations were unnecessary. Younis *et al.* [10] in his research showed a zero prevalence of malignancy among the 2,099 Paediatric patients undergoing tonsillectomy but noted that this incidence differed from the adult population [10].

A lot of money goes into biopsies of adeno-tonsillar tissues sent for histology. From our study, a histology of just either an adenoid or tonsillar tissue costs about 9000 or 26 US Dollars. This is even higher in a study conducted by Kalcioglu *et al.* [11] where histology examinations of tonsil vary ranging from US $12.85 to US $90.00. In Brazil it is about R$20.03. In addition, DellAringa *et al*. in his study, also noted a negative cost-benefit ratio for routine histopathologic exams [12] Indeed with a thorough physical examination and radiology findings and decent adeno-tonsillectomy, our nation could save billions of Naira per annum if histology of adeno-tonsillar specimen is avoided. Cost-benefit analysis (CBA), often called benefit costs analysis (BCA), is a systematic approach to estimate the strengths and weaknesses of alternatives; it is also used to determine options that provide the best approach to achieve benefits while preserving savings [13]. The final step in CBA is to quantitatively compare the results of the aggregate costs and benefits to determine if the benefits outweigh the costs. If so, then the rational decision is to go ahead with the project. The CBA is also defined as a systematic process for calculating and comparing benefits and costs of a decision, policy [14]. In other words, adeno-tonsillar diseases are an important health problem, leading to large numbers of surgical procedures worldwide [15]. Also, routine histological examination of tonsillectomy and adenoidectomy specimens is performed in many parts of the world so as not to miss rare but significant pathological findings [16].

In South Africa, routine histological study of tonsillectomy and adenoidectomy specimens has a low cost-benefit rate, although, due to legal and ethical issues, physicians may request routine histological examination [17]. Most otolaryngology services routinely send adeno-tonsillectomy specimens for histopathologic examination, whether for malignancy investigation, analysis of suspect material, or medico-legal documentation of surgical removal [8]. But recent studies have shown that routine histopathologic analysis of the tonsil is dispensable, because they have a very low probability of diagnosing occult malignancies [7]. Unfortunately, this risk is still not zero, so the need for routine histopathology is still controversial [7] given the low incidence of occult malignancy in the absence of acknowledged risk factors, routine histologic evaluation of adeno-tonsillectomy specimens is not recommended. Discontinuation of regular histologic evaluation would result in an annual United States cost savings of approximately $35,467,080 [18]. Furthermore, Van Lierop and Prescott carried out a cost-benefit analysis on 172 children in South Africa, by multiplying the cost of histological examination of a tonsillectomy and adeno-tonsillectomy specimen by the number of specimens sent [16]. Using the same method for this current study, the CBA involved in conveyance of adeno-tonsillar tissues for histology, will cost 9000 Naira (26 US dollars) for each specimen, therefore for 72 specimens used in this study, the total amount of money spent in conducting the histology is about 648,000 Naira (1851.4 US dollars)., Conclusively, due to the high cost involved in passage of adeno-tonsillar tissues for histology, it is dispensable [18].

## Conclusion

Routine histopathologic examination in adeno-tonsillectomy specimens among children is dispensable, with a negative cost-benefit ratio.

### What is known about this topic

- A lot of money is spent pursuing histology on adenoid tissues of children who underwent adeno-tonsillectomy and yet the results always show normal adeno-tonsillar tissue.

### What this study adds

- This study shows that there is a zero prevalence of malignant transformation on all adenoid tissue sent for histology. This implies that routine biopsies of adeno-tonsillar specimens pose a negative cost-benefit ratio. There is therefore no need paying so much for histology of adenoid tissue that will definitely show no malignant transformation.

## Competing interests

The authors declare no competing interests.

## References

[1] Wysocka J, Hassmann E, Lipska A, Musiatowicz M (2003). Naïve and memory T cells in hypertrophied adenoids in children according to age. Int J Pediatr Otorhinolaryngol.

[2] Yildrim N, Sahan M, Karsliglu Y (2008). Adenoid hypertrophy in adults: clinical and morphological characteristics. J Int Med Res.

[3] Manas R, Diganta M, Vijaylaxmi Y, Kamlesh B, Chakradhar M (2013). Adenoid Hypertrophy in Adults: a case Series. Indian J Otolaryngol Head Neck Surg.

[4] Kamel RH, Ishak EA (1990). Enlarged adenoid and hypertrophy in adults: endoscopic approach and hisopathological study. J Laryngol Otol.

[5] Yong-sheng Z, Wan-jun Z (1989). A morphologic and follow-up study on the nasopharyngeal lymphoid hyperplasia and its relation to cancer. Chin Med J.

[6] Giseli R, Thiago EP, Elias LB, Willian MM, Fernando R, Cícero M (2013). Are histologic studies of adenotonsillectomy really necessary?. Int Arch Otorhinolaryngol.

[7] Verma SP, Stoddard T, Gonzalez-Gomez I, Koempel JA (2009). Histologic analysis of pediatric tonsil and adenoid specimens: is it really necessary?. International Journal of Pediatric Otorhinolaryngology.

[8] Randall DA, Martin PJ, Thompson LD (2007). Routine histologic examination is unnecessary for tonsillectomy or adenoidectomy. Laryngoscope.

[9] Garavello W, Romagnoli M, Sordo L, Spreafico R, Gaini RM (2004). Incidence of unexpected malignancies in routine tonsillectomy specimens in children. Laryngoscope.

[10] Younis RT, Hesse SV, Anand VK (2001). Evaluation of the utility and cost-effectiveness of obtaining histopathologic diagnosis on all routine tonsillectomy specimens. Laryngoscope.

[11] Kalcioglu MT, Gurses I, Erdem T (2010). Is the pathological examination of routine tonsillectomy and adenoidectomy specimens necessary? A retrospective study of 559 adenoidectomy and 1132 tonsillectomy specimens and a literature review. B-ENT.

[12] Dell'Aringa AR, Juares AJ, Melo CD, Nardi JC, Kobari K, Perches Filho RM (2005). Histological analysis of tonsillectomy and adenoidectomy specimens-January 2001 to May 2003. Braz J Otorhinolaryngol.

[13] David R, Ngulube P, Dube A (2013). A cost-benefit analysis of document management strategies used at a financial institution in Zimbabwe: a case study. South African Journal of Information Management.

[14] Hemakumara GPTS (2017). Cost-benefit analysis of proposed godagama development node under the greater Matara Development Planning Program. International Research Journal of Management and Commerce.

[15] Proenca-Modena JL, Pereira Valera FC, Jacob MG, Buzatto GP, Saturno TH, Lopes L (2012). High Rates of Detection of Respiratory Viruses in Tonsillar Tissues from Children with Chronic Adenotonsillar Disease. PLoS ONE.

[16] Van Lierop AC, Prescott CAJ (2009). Is routine pathological examination required in South African children undergoing adenotonsillectomy. South African Medical Journal.

[17] Alfredo R, Dell'Aringa JC, Juares CC, Nardi K, Renato MP (2005). Histological analysis of tonsillectomy and adenoidectomy specimens. Brazilian Journal of Otorhinolaryngology.

[18] Robert HF (2000). Why is cost-benefit analysis so controversial. The Journal of Legal Studies.

